# Altered microRNAs expression profiling in cumulus cells from patients with polycystic ovary syndrome

**DOI:** 10.1186/s12967-015-0605-y

**Published:** 2015-07-22

**Authors:** Suying Liu, Xuan Zhang, Changgen Shi, Jimin Lin, Guowu Chen, Bin Wu, Ligang Wu, Huijuan Shi, Yao Yuan, Weijin Zhou, Zhaogui Sun, Xi Dong, Jian Wang

**Affiliations:** Department of In-Vitro Fertilization, Shanghai Zhongshan Hospital, Shanghai, China; National Populations and Family Planning Key Laboratory of Contraceptive Drugs and Devices, The Shanghai Institute of Planned Parenthood Research (SIPPR), Shanghai, China; Institute of Biochemistry and Cell Biology, Shanghai Institute for Biological Sciences, Chinese Academy of Sciences, Shanghai, China; Shanghai Key Laboratory of Female Reproductive Endocrine-Related Diseases, Shanghai Jiai Genetics and IVF Institute, Obstetrics and Gynecology Hospital of Fudan University, Shanghai, China; Medical School of Fudan University, Shanghai, China

**Keywords:** Polycystic ovary syndrome, MicroRNAs, Cumulus cells, Expression profile

## Abstract

**Background:**

Polycystic ovary syndrome (PCOS) is a common endocrine disorder in women of reproductive age, and oocyte developmental competence is altered in patients with PCOS. In recent years microRNAs (miRNAs) have emerged as important regulators of gene expression, the aim of the study was to study miRNAs expression patterns of cumulus cells from PCOS patients.

**Methods:**

The study included 20 patients undergoing in vitro fertilization (IVF) and intra-cytoplasmic sperm injection (ICSI): 10 diagnosed with PCOS and 10 matching controls. We used deep sequencing technology to identify the miRNAs differentially expressed in the cumulus cells of PCOS.

**Results:**

There were 17 differentially expressed miRNAs in PCOS cumulus cells, including 10 miRNAs increase and 7 miRNAs decrease. These miRNAs were predicted to target a large set of genes with different functions, including Wnt- and MAPK- signaling pathways, oocyte meiosis, progesterone-mediated oocyte maturation and cell cycle. Unsupervised hierarchical clustering analysis demonstrated that there was a specific miRNAs expression pattern in PCOS cumulus cells.

**Conclusion:**

We found that the miRNAs expression profile was different in cumulus cells isolated from PCOS patients compared with control. This study provided new evidence for understanding the pathogenesis of PCOS.

## Background

Polycystic ovarian syndrome (PCOS) is a very common female endocrine disorder among infertile female individuals and affects approximately 5–10% of women of reproductive age worldwide. Women with PCOS frequently present with reproductive dysfunction, and PCOS is the main cause of infertility due to dysfunctional follicular maturation and ovulation, distinctive multicystic ovaries and dysregulation of reproductive hormones including luteinizing hormone (LH) hypersecretion and hyperandrogenism. Besides the reproductive abnormalities, PCOS women also exhibit non-reproductive metabolic abnormalities such as obesity, metabolic syndrome, hyperinsulinemia, insulin resistance, dyslipidemia and type 2 diabetes [1–4]. Despite its prevalence, the etiology of PCOS remains unclear.

Although it is difficult to define the exact pathogenesis of anovulation in PCOS, many possible mechanisms have been postulated. Hypersecretion of LH, hyperandrogenaemia and hyperinsulinaemia have all been investigated as possible causes of PCOS. It has been showed that alteration of many factors might directly or indirectly impair the competence of maturating oocytes through endocrine and local paracrine/autocrine actions, resulting in a lower pregnancy rate in patients with PCOS [5–8]. It was likely that these factors were interlinked together and might result in disordered ovarian function in PCOS [9].

In recent years, miRNAs have emerged as critical regulators of gene expression in mammals. The non-coding RNA molecules serve as a fundamental role in the regulation of gene expression, and it is estimated that the expression of 30% of genes are the potential target of miRNAs regulatory function [10]. By using Dicer1 conditional knockout (cKO) mouse, people showed that Dicer1, the ribonuclease III for synthesis of mature functional miRNAs, played important roles in follicular cell development through the differential regulation of gene expression [11]. Recently, it was reported that miR-29a, miR-30d and miR-224 were involved in folliculogenesis in mouse [12].

Folliculogenesis in anovulatory women with PCOS is characterized by failure of dominance and the ovary has multiple arrested small follicles. The ovarian follicular microenvironment and maternal signals were responsible for oocyte growth, development and the gradual acquisition of oocyte developmental competence [13]. The cumulus cells are a subset of granulose cells which maintain an intimate connection with the oocyte and are responsible for providing several tropic and metabolic factors to the pre-ovulatory oocyte. It has been found that cumulus cells played an important roles in promoting normal cytoplasmic maturation of oocytes necessary for pronuclear formation and subsequent development capability [14].

Because cumulus cells can obtained easily before intra-cytoplasmic injection (ICSI) procedure or before insemination for classical in vitro fertilization (IVF), several groups used microarray technologies, RT-PCR and quantitative RT-PCR analyses to link the cumulus cells gene expression profile with oocyte quality and/or embryo competence and/or pregnancy outcome [5, 15–17]. In recent years, there were some studies about the transcriptional profiles of human cumulus cells from PCOS patients [18–21]. Although a large number of genes have been shown to be associated with PCOS, the underlying post-transcriptional regulation of gene expression remains poorly understood [18, 22–25], especially the role of miRNAs in human cumulus cells [26].

Several features of miRNAs, including their stability and tissue specificity, make them suitable for their detection and relative quantification in a variety of tissues. Genomic scale profiling has been routinely applied in miRNAs research. Deep sequencing, which utilizes the latest massively parallel sequencing, has provided an alternative way to obtain miRNAs profiles at unprecedented sensitivity [27, 28]. This study was aimed at the identification and quantification of miRNAs in PCOS cumulus cells, and in order to investigate the function miRNAs in PCOS.

## Methods

### Participants and samples

Samples were collected after the patients gave their written informed consent and the study was approved by the Ethical Committee of the The Obstetrics and Gynecology Hospital of Fudan University (Shanghai Red House Ob and Gyn Hospital) and carried out in compliance with the Helsinki Declaration.

The diagnosis of PCOS was based on the Rotterdam revised criteria after ruling out secondary causes [29]. Between July 2012 and December 2012, a total of 20 participants (10 PCOS and 10 controls) were enrolled in the study. All patients had no history of drugs affecting glucose and lipid metabolism, and inclusion criteria for all of the subjects included the following: a basal follicle stimulating hormone (FSH) level of <10 IU/L; age <35 years; a body mass index (BMI) <25 kg/m^2^ (Table 1). We measured the level of FSH and LH on day 3 of menstrual cycle before the ovarian stimulation. Exclusion criteria in control included any known medical conditions or diseases. Care was taken to exclude hyperandrogenism and chromic anovulation.

**Table 1:** Characteristic of PCOS and control participants

Characteristic	Control (n = 10)	PCOS (n = 10)
Age (years)	29.4 ± 3.0	27.4 ± 2.6
BMI (kg/m^2^)	23.5 ± 3.2	22.0 ± 3.5
FSH (mIU/ml)	7.5 ± 2.5	5.2 ± 1.6
LH (mIU/mL)	4.3 ± 1.6	7.3 ± 1.7
LH/FSH	0.57 ± 0.2	1.4 ± 0.6
Oocytes obtained	8.8 ± 3.0	18.8 ± 5.3
No of MII oocytes	6.6 ± 2.0	15.6 ± 7.7
2PN	5.8 ± 1.4	14.6 ± 6.3
No of transferable embryos	4.2 ± 2.0	8.8 ± 4.9

All the participants included in the study were women undergoing IVF with ICSI at Shanghai Jiai Genetics and IVF Institute, and all the ICSI cycles included were conducted according to the long mid-luteal GnRH agonist (Ferring Pharmaceuticals, Switzerland) protocol. All patients were undergoing ICSI due to male factor infertility. Controlled ovarian stimulation was conducted with recombinant human FSH (Gonal-f, Merck Serono). Oocytes injection and embryos culture were performed as described previously [30]. For oocyte retrieval, all patients underwent ovarian puncture of follicles >15 mm in diameter. Only the cumulus-oocyte complexes with mature oocytes (MII) were included in this study.

### Cumulus cell collection

The cumulus cells were collected as previously described [31]. Briefly, oocyte retrieval was performed transvaginally 35 h after the triggering of ovulation. Cumulus-oocyte complexes were collected in MOPS medium (Vitrolife, Sweden) and the cumulus cell were mechanically stripped from oocyte after brief exposure to hyaluronidase (Vitro-life, Sweden) under stereomicroscopy. The cumulus cells were washed three times with culture medium and frozen individually prior to total RNA extraction.

### Small RNA deep sequencing and data analyses

We randomly selected 5 PCOS and 5 control samples used for deep sequencing. Total RNA from cumulus cells of each sample was extracted using TRIzol reagent according to the manufacturer’s protocol (Invitrogen, USA), respectively. The concentration of the RNA was measured by NanoDrop and the RNA integrity was checked on Bioanalyzer2100 (Agilent, USA). 200 ng of total RNA was used for preparing small RNA libraries for deep sequencing according to the manufacturer’s instructions (Illumina, USA). The cDNA libraries were sequenced on Illumina HiSeq 2000 instrument with 50-base pair single reads. Raw sequencing data was mapped to human miRNA database (miRBase v21) using Bowtie2.

The Mann–Whitney test was performed to discover differentially expressed miRNAs between PCOS and control samples. The miRNAs used for biological functional analyses were significantly differentially expressed (*p* < 0.05 and fold change >2) in the cumulus cells of PCOS.

Unsupervised hierarchical clustering analysis was conducted. We perform cluster analysis of gene expression patterns with “gplots”, which is a package for R. (http://CRAN.R-project.org/package=gplots) software. We used the Euclidean distance measure and the average linkage clustering algorithm. The branching pattern was illustrated in a dendrogram in which the similarity between the miRNAs expression profiles could be visually assessed.

To determine miRNAs regulation pathway, we searched for putative miRNAs that are able to target based on commonly cited prediction programs such as miRanda [32]. GO term was assigned to each target gene according to Gene Ontology, and then ultra-geometric test was used to find out enrich term in this analysis. In the same way, pathway id was assigned to every gene by KEGG database, then ultra-geometric test was used to find out enrichment pathway in our analysis.

### Real-time PCR

After random selection 5 PCOS and 5 control samples used for deep sequencing, the remaining samples were used for real-time PCR. Total RNAs of 10 samples were extracted using TRIzol reagent (invitrogen), respectively. RT-qPCR was carried out using the miRCURY LNA™ Universal RT miRNAs PCR system (Exiqon, MA, USA) according to the manufacturer’s description, and analysed using an ABI 7900 HT (Applied Biosystems, Foster, CA, USA). All miRNAs Assay primers used in this study were purchased commercially (Exiqon, MA, USA). Primer efficiencies were determined by standard curve. Relative miRNAs expression was calculated by efficiency-corrected ΔΔC_t_ method, normalized to the endogenous control U6 snRNA. Each sample in each group was measured in triplicate and the experiment was repeated for at least three times.

### Statistical analysis

All values are presented as mean ± SEM. Statistical comparisons among groups were analyzed by one-way ANOVA followed by student’s t test using SPSS software package (version 10.0.1, SPSS Inc., Chicago, IL, USA). A value of *p* < 0.05 was considered significant.

## Results

### Differentially expressed miRNAs profiles of cumulus cells isolated from PCOS and control

By deep sequencing, we have obtained a total of 29,955,439 sequences reads, and 5,448,483 sequences reads of 648 kinds of miRNAs, which perfectly matched human miRNAs database (miRBase v21). Then the Mann–Whitney test was performed to discover differentially expressed miRNAs between PCOS and control group. We focused on miRNAs meeting our designated criteria: *p* < 0.05 and fold change ≥2. A total of 17 miRNAs were identified: 10 miRNAs upregulated and 7 miRNAs down-regulated on PCOS compared with control (Table 2).

**Table 2:** Differentially expressed miRNAs in PCOS

miRNA name	PCOS	Control	Folds change	*P* value
hsa-miR-513a-3p	49	9	5.6	0.009
hsa-miR-508-3p	181	69	2.6	0.009
hsa-miR-513b	20	6	3.4	0.009
hsa-miR-514	288	84	3.4	0.009
hsa-miR-509-5p	108	33	3.3	0.009
hsa-miR-513c	13	4	3.5	0.011
hsa-miR-151-3p	135	216	0.6	0.028
hsa-miR-144	45	7	6.2	0.028
hsa-miR-510	36	16	2.2	0.028
hsa-miR-720	32	88	0.4	0.028
hsa-miR-615-3p	17	58	0.3	0.028
hsa-miR-509-3p	551	255	2.2	0.028
hsa-miR-127-3p	361	1,045	0.3	0.028
hsa-miR-455-3p	11	25	0.4	0.036
hsa-miR-342-3p	85	172	0.5	0.036
hsa-miR-654-3p	20	40	0.5	0.046
hsa-miR-508-5p	38	20	1.9	0.047

**Figure 1:**
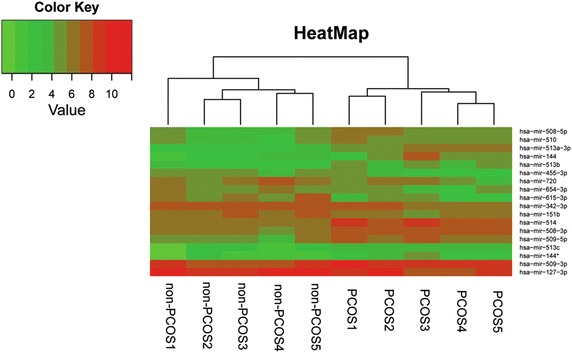
Unsupervised hierarchical clustering of deep sequencing data. Heatmap of miRNA expression data after hierarchical clustering. The Euclidean distance measure and the average linkage clustering algorithm were used. The branching pattern was illustrated in a dendrogram.

To gain a systematic comparison between the two groups of miRNAs expression profiles, unsupervised hierarchical clustering analysis was conducted. The unsupervised hierarchical clustering generated a tree with a clear distinction of samples in two main groups, represented by PCOS and control (Figure 1).

**Figure 2:**
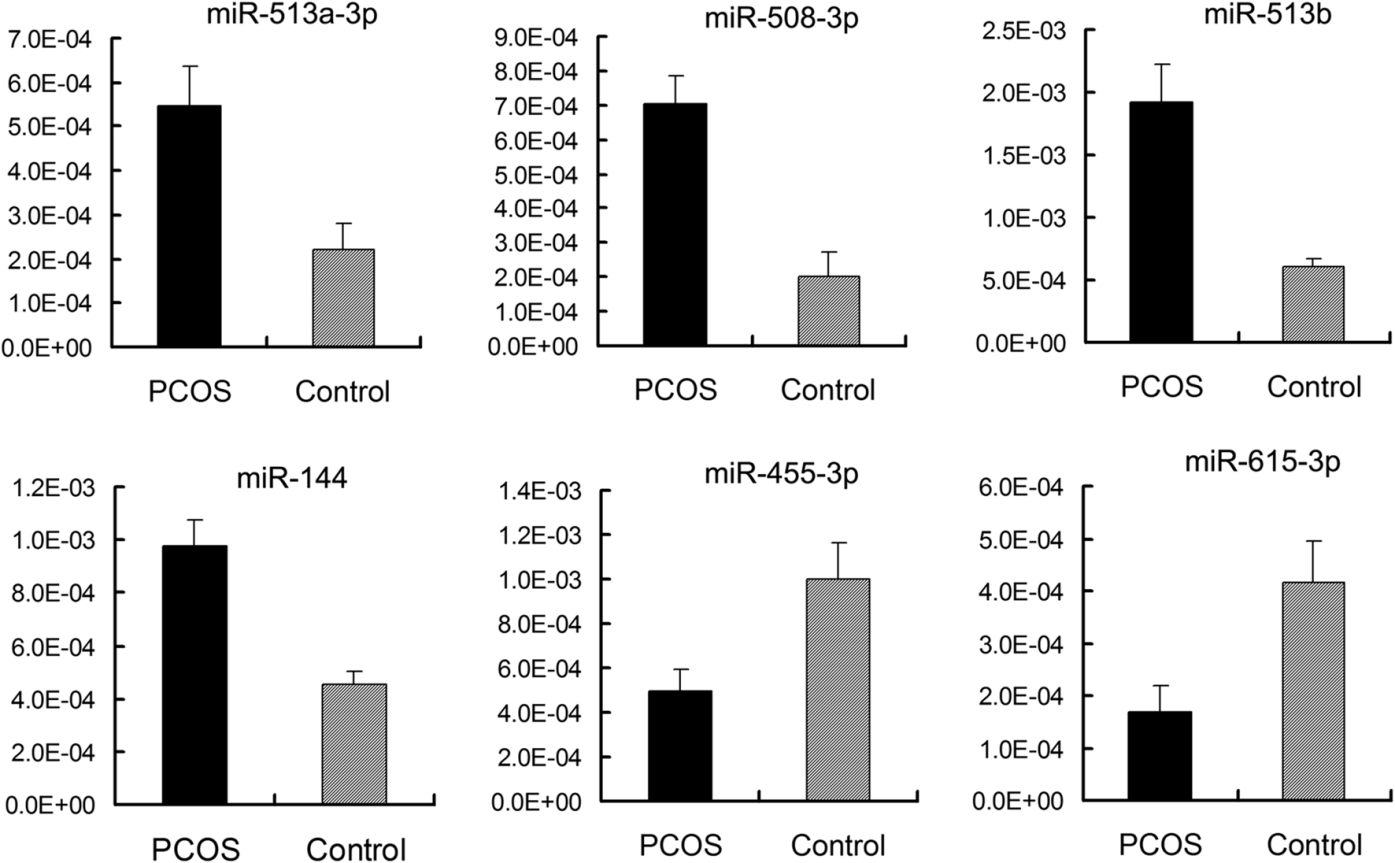
Differential expression of six miRNAs in cumulus cells of PCOS patients and control.

To validate the reliability of the deep sequencing data, we selected 6 miRNAs, 4 up-regulated miRNAs (hsa-miR-513-3p, hsa-miR-508-3p, hsa-miR-513b and hsa-miR-144) and 2 down-regulated miRNAs (hsa-miR-455-3p and hsa-miR-615-3p) in PCOS, confirmed that their expression pattern using qPCR. The results showed that the expression pattern of all 6 miRNAs analyzed by qPCR were in concordance with the deep sequencing data, which indicated that our deep sequencing data were reliable (Figure 2).

### Identification and functional analysis of miRNA-predicted targets

**Figure 3:**
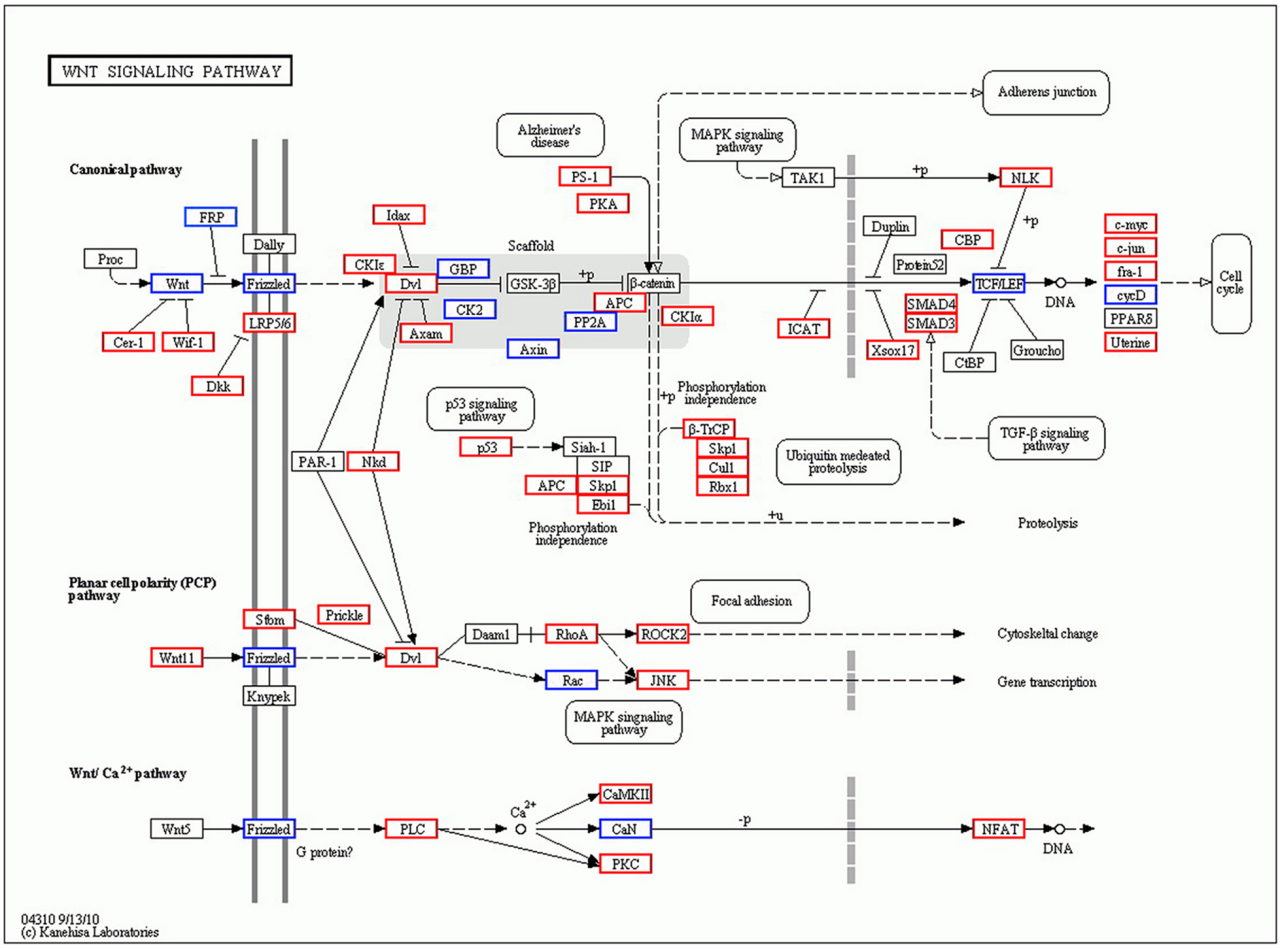
Transcripts regulating the Wnt signaling pathway are putatively affected by up-regulated miRNAs expression in the PCOS patients when compared with control. *Red boxes* represent transcripts affected by only one miRNAs, whereas *blue boxes* indicate transcript affected by several miRNAs. *White boxes* indicate genes belonging to the Wnt-signaling pathways that showed no differential expression between the two groups. The diagram is based on the Wnt signaling pathway in the kyoto encyclopedia of genes and genomes pathway database.

The predicted target mRNAs of the 17 differentially expressed miRNAs were identified in silico. Using a computational miRNAs target gene prediction (miRanda), we identified 3781 putative differentially expressed mRNAs. The functions of the predicted miRNAs target gene and the molecular pathways they potentially constitute were assessed using GO and KEGG analysis. The predicted targets were significantly enriched for several biological functions known to be involved in oocyte development, mitogen activated protein kinase (MAPK) signaling pathway, Wnt signaling pathway, regulation of actin cytoskeleton, steroid hormone biosynthesis, oocyte meiosis, gap junction and GnRH signaling pathway (Table 3). The Wnt signaling network enriched for miRNAs targets was shown in Figure 3. We chose to present this particular pathway since this pathway converges with other pathways that biologically relevant to oocyte development, such as cell cycle, adherens junction and transforming growth factor beta (TGF beta).

**Table 3:** Annotation results from GO analysis

	*P* value	Ratio	
Pathways			
MAPK signaling pathway	1.21E−60	160	287
Wnt signaling pathway	4.87E−49	104	159
Insulin signaling pathway	1.93E−20	90	230
Notch signaling pathway	1.09E−18	36	53
p53 signaling pathway	3.02E−18	46	83
GnRH signaling pathway	3.66E−14	60	153
Hedgehog signaling pathway	1.03E−13	35	65
Networks			
Regulation of actin cytoskeleton	1.91E−30	125	303
Focal adhesion	1.34E−26	124	324
Cell cycle	8.90E−23	87	204
Oocyte meiosis	1.55E−19	70	159
ECM-receptor interaction	3.62E−19	57	115
Gap junction	3.09E−16	61	144
Pancreatic secretion	2.98E−12	61	171
Progesterone-mediated oocyte maturation	1.66E−10	52	147
Diseases			
Pancreatic cancer	8.85E−14	42	88
Type I diabetes mellitus	1.28E−11	30	57
Endometrial cancer	1.56E−09	31	70
Type II diabetes mellitus	4.05E−08	24	52
Maturity onset diabetes of the young	8.60E−08	17	30
Metabolisms			
Glycosaminoglycan biosynthesis-heparan sulfate	6.85E−17	23	26
N-Glycan biosynthesis	5.31E−16	32	49
Starch and sucrose metabolism	4.34E−13	33	61
Amino sugar and nucleotide sugar metabolism	9.32E−09	24	49
Fructose and mannose metabolism	2.38E−08	22	44
Glycine, serine and threonine metabolism	4.03E−08	22	45
Galactose metabolism	6.36E−07	16	30
Steroid biosynthesis	9.98E−05	10	19

## Discussion

In this study, cumulus cells from PCOS and control were collected before ICSI, and miRNAs expression profiles were analyzed using deep sequencing technology. We detected 648 known miRNAs in cumulus cells. Comparing miRNAs expression profiles between PCOS and control group, we identified that there were 17 miRNAs differentially expressed. To confirm the reliability of the deep sequencing data, we selected 6 miRNAs to examine their expression pattern in cumulus cells of PCOS and controls by using qPCR. The results of qPCR were consistent with deep sequencing data. Unsupervised hierarchical clustering analysis showed a separation between PCOS and control group. Investigating the role of miRNAs in PCOS, Xu et al. found that a total of 59 known miRNAs were differentially expressed in PCOS cumulus granulosa cells, including 21 miRNAs increase and 38 miRNAs decrease [33]. Comparing our results with that of the previous results [33], we found that there was no overlap of the differentially expressed miRNAs at all. The inconsistency of results obtained from our lab with the results obtained from Xu’s might reinforce the finding that PCOS is a multi-factorial and heterogeneous syndrome. So, identification of differentially expressed miRNAs in a larger scale of patients may help to improve understanding of the underlying molecular mechanism of PCOS.

Since each miRNA has been predicted to have a broad range of target mRNA based on the degree of sequence homology. We would like to identify their predicted targets as well as the molecular networks and the biological functions they may affect. To fully characterize these differently expressed miRNAs, gene ontology analysis was performed on the predicted gene targets for the 17 differentially regulated miRNAs, which putatively regulate the expression of 3781 genes. It was revealed that these genes associated with diverse important signaling pathways such as MAPK signaling pathway, Wnt signaling pathway, insulin signaling pathway and GnRH signaling pathway. Our results was supported by the finding that the genes of the Wnt- and MAPK-signaling pathways were generally down-regulated in the PCOS [19]. Importantly, the predicted gene targets were associated with oocyte meiosis and progesterone-mediated oocyte maturation. Also, the gene ontology analysis results predicted that the differentially expressed miRNAs might involved in Type I diabetes mellitus, Type II diabetes mellitus, starch and sucrose metabolism and steroid biosynthesis (Table 3). So, our results suggested that miRNAs might play important roles in PCOS, and elucidating the roles of miRNAs in PCOS should be the subject of future investigations.

The changes in the expression of even a single miRNA could have a detrimental impact on the outcome of diverse cellular activities regulated by the product of these genes, such as oocyte development and maturation. Because these dysregulated genes might link to diverse pathways such as lipid metabolism, and insulin signaling and oocyte maturation, which was proven related to PCOS, we speculated that differently expressed miRNAs might be involved in follicular growth arrest and metabolic disorders associated with PCOS.

Several studies on miRNAs expression have been done on the different ovarian components of human, such as granulose cells [34] and follicular fluid [35, 36], but the possible role of miRNAs within the pathophysiology of PCOS has only been sparsely investigated [12, 34, 35, 37–39]. Little was known regarding the involvement of miRNAs during follicular development and in PCOS. In PCOS, an accelerated early follicular growth leaded to an excess of small follicles [40]. Different factors have been reported to participate in the growth of follicles and the maturation of oocyte [41]. The TGF beta superfamily, which includes inhibins, activins, growth differentiation factors (GDFs) and bone morphogenetic protein (BMPs), played a central role in many processes that governed follicle development, oocyte maturation and competence [42]. It has been reported that in PCOS cumulus cells, 65% of genes related to the TGF beta signalling pathways were down-regulated, including several members of the TGF beta superfamily, type II TGF beta receptors and their targets SMAD1/5, as well as the TGF beta receptor III (TGFBR3) [18]. According to our heatmap, hsa-miR-514, hsa-miR-144 and hsa-miR-513a-3p differentially expressed in PCOS patients. When classified into biological function, these three miRNAs were enriched for several pathways known to be crucial in the process of oocyte development and maturation such as TGF-beta signaling pathway, MAPK signaling pathway, estrogen signaling pathway and oocyte maturation, etc. The functional annotation of differentially expressed miRNAs in our study was in agreement with other studies that have found that defects in the TGF-beta and estrogen receptors signalling cascades may contribute to the reduced oocyte developmental competence in PCOS [18, 26]. Also, miR-513a-3p and luteinizing hormone/chorionic gonadotropin receptor (LHCGR) were both expressed in human ovarian granulosa cells. The LHCGR is a G protein-coupled receptor (GPCR) that plays a central role in ovarian follicular maturation, ovulation and maintenance of corpus luteum and pregnancy. LHCGR was a predicted target of miR-513a-3p, and downregulation of LHCGR expression by miR-513a-3p has been observed [43]. Therefore, miR-513a-3p may be involved in follicular maturation in response to abnormal hormone microenvironment in PCOS follicles.

Oocyte competence depends on the quality of the follicular microenvironment and the presence of adequate bidirectional cumulus cell-oocyte signaling is primordial for both oocyte and cumulus cells competence acquisition [20, 44]. Interaction between cumulus cells and oocytes involves both gap junctions and paracrine signaling factors. Gap junctions allow transfer of small molecules and facilitate exchange of glucose metabolites and ions between the cumulus cells and oocytes [45]. Our results found that hsa-miR-508-5p/3p, when classified into biological function, were enriched for several pathways known to be crucial in cell communication, including ECM-receptor interaction, focal adhesion, tight junction and gap junction. Our miRNAs biological function prediction results implied that miRNAs might involved in cell communication system during oocyte competence development, but the molecular mechanism still need be identified.

## Conclusions

In summary, we identified differentially expressed miRNAs in PCOS cumulus cells. Bioinformatics analysis gene targets of these differentially expressed miRNAs provided additional information on the molecular events that lead to PCOS pathogenesis. Many of the miRNA-regulated molecular networks and biological processes identified here corroborated what was previously suggested in the literature about oocyte competence defects. The molecular pathways presented here constituted a comprehensive resource on which to base future investigations into the role of specific miRNAs in PCOS.

## Abbreviations

BMP: bone morphogenetic protein
cKO: conditional knockout
ICSI: intra-cytoplasmic injection
IVF: in vitro fertilization
LH: luteinizing hormone
LHCGR: luteinizing hormone/chorionic gonadotropin receptor
miRNAs: microRNAs
MAPK: mitogen activated protein kinase
GDF: growth differentiation factor
GPCR: G protein-coupled receptor
PCOS: polycystic ovary syndrome
TGF: transforming growth factor
